# A Case Report of a Giant Basilar Artery Aneurysm

**DOI:** 10.7759/cureus.51018

**Published:** 2023-12-24

**Authors:** Ahlam Alharbi, Meshal Alharbi, Mohammed Alharbi, Faisal Almishali, Hatem Alzhrani, Jumana Al-Najaidi, Manal Aljohani, Sara Sabba, Layla Abdulla, Mahmood Alaiwi, Mohamed Hasan, Alhanouf Hatim, Eman Abdulla, Abdulqadir Maddah

**Affiliations:** 1 Family Medicine, Primary Health Care Center, Riyadh, SAU; 2 General Practice, Qassim University, Qassim, SAU; 3 General Practice, Al-Rayan Colleges, Riyadh, SAU; 4 General Practice, University of Rostock, Rostock, DEU; 5 General Practice, Al-Baha University, Al-Baha, SAU; 6 General Practice, Taif University, Taif, SAU; 7 General Practice, Wenzhou Medical University, Wenzhou, CHN; 8 General Practice, First Moscow State Medical University, Moscow, RUS; 9 General Practice, Umm Al-Qura University, Mecca, SAU; 10 General Practice, Mansoura University, Mansoura, EGY; 11 Anesthesia and Critical Care, Dallah Hospital, Riyadh, SAU

**Keywords:** magnetic resonance angiography, endovascular procedures, aneurysm, basilar artery, chronic headache

## Abstract

Intracranial aneurysms, characterized by the localized dilation of cerebral arteries, pose a substantial risk of rupture, leading to severe consequences. Basilar artery aneurysms, in particular, present unique challenges due to their location and potential impact on vital brainstem structures. Advanced diagnostic imaging has improved the chances of early identification of the condition, enabling timely intervention. We discuss the case of a 54-year-old female with controlled hypertension, who presented with persistent severe headaches and neurological symptoms. Diagnostic investigations revealed a large saccular basilar artery aneurysm measuring 4.7 cm. The aneurysm exerted pressure on the brainstem. After comprehensive discussions, the patient underwent successful flow-diverter stent placement, which led to the gradual resolution of symptoms. The multidisciplinary team closely monitored the patient in the neurointensive care unit. Managing giant basilar artery aneurysms poses significant challenges due to the potentially life-threatening complications associated with it. The success in treating the presented case underscores the importance of a multidisciplinary approach involving neurosurgery, interventional radiology, and critical care in managing these patients.

## Introduction

Intracranial aneurysms represent a complex and clinically significant pathology among cerebrovascular disorders. Characterized by the localized dilation of cerebral arteries, these vascular anomalies pose a substantial risk of rupture, leading to devastating consequences such as subarachnoid hemorrhage. Its prevalence is estimated to be approximately 3% in the general population [1]. While various risk factors, including hypertension and genetic predisposition, have been implicated in their development, the nuanced interplay between genetic and environmental factors remains a subject of ongoing research [2].

A subset of aneurysms involving the basilar artery presents unique challenges due to their location and potential impact on vital brainstem structures. Advanced diagnostic imaging techniques have enabled the early identification and characterization of intracranial aneurysms, leading to improved patient outcomes through timely intervention [3]. We discuss a case of a 54-year-old female with a history of well-controlled hypertension, who presented with persistent severe headaches and associated neurological symptoms. Subsequent diagnostic investigations revealed a large saccular basilar artery aneurysm.

## Case presentation

A 54-year-old female, with a history of well-controlled hypertension, presented to the neurology clinic with a three-month history of persistent, severe headaches, occasionally accompanied by nausea and photophobia. The patient had no family history of neurological disorders. Initial vital signs were within normal limits, and a thorough neurological examination revealed no focal deficits, cognitive impairment, or signs of meningeal irritation. Fundoscopic evaluation showed no papilledema. Given the persistence and intensity of the headaches, a comprehensive diagnostic workup was initiated. Laboratory investigations, including a complete blood count and a basic metabolic panel with normal electrolyte levels, were conducted to rule out other potential causes.

**Figure 1:**
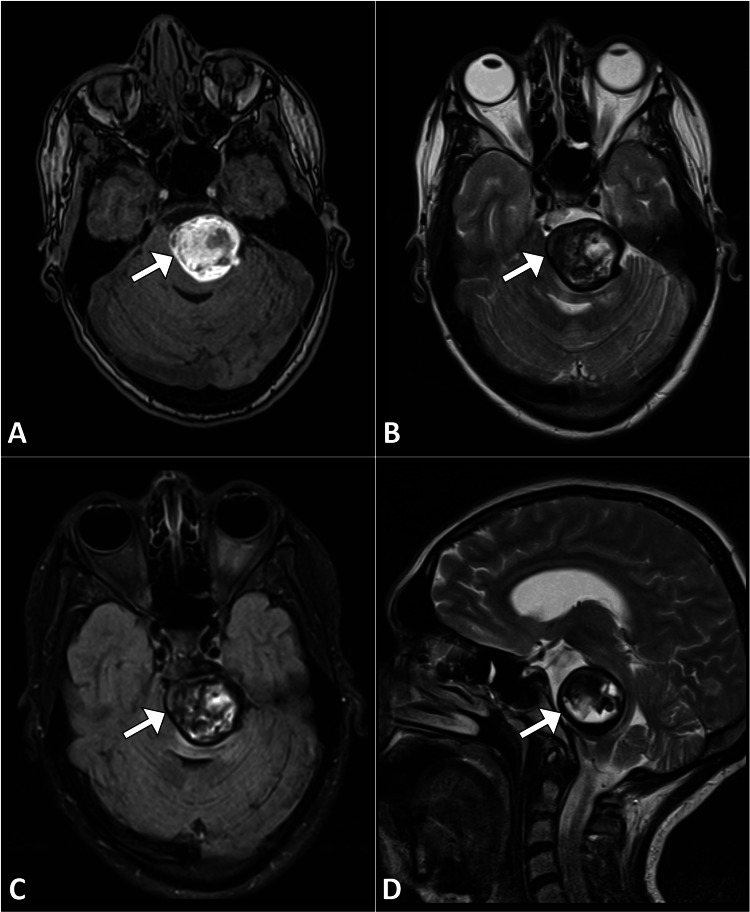
Axial (A-C) and sagittal (D) MR images depict the basilar artery's aneurysmal dilatation (arrow) with partial thrombosis, as observed through T1-weighted image (A), T2-weighted image (B), and FLAIR sequence (C) MR: magnetic resonance; FLAIR: fluid-attenuated inversion recovery

In light of the symptoms and the need to explore potential structural anomalies, an MRI of the brain was performed, which revealed a saccular basilar artery aneurysm measuring 4.7 cm in the largest diameter, located in the mid part of the basilar artery. The aneurysm demonstrated a characteristic saccular morphology with an intact, yet irregular, wall. Within the aneurysm, a non-occlusive thrombus was identified. There was evidence of a significant pressure effect on the brainstem, manifesting as marked narrowing of the pons and medulla oblongata. Surrounding vascular anatomy appeared patent with normal caliber. The aneurysmal wall did not show any signs of rupture (Figure 1).

The patient was referred to a specialized center for further evaluation and management. The management approach at the center involved collaboration among a multidisciplinary team comprising neurosurgeons, interventional radiologists, and neurologists. We decided to treat the aneurysm endovascularly despite its wide neck since the patient had refused any surgical intervention. Due to the aneurysm configuration and size, endovascular treatment alone by coiling appeared dangerous. Hence, we employed a comprehensive strategy by placing a flow-diverter stent in the basilar artery to create a new and safe lumen for the parent artery. Subsequently, through the previously positioned microcatheter in the aneurysm sac, we filled the aneurysm sac with coils.

Before the procedure, the patient had been on clopidogrel, and during the intervention, we administered heparin to prevent thromboembolic complications. The procedure, conducted under general anesthesia, involved the placement of the flow-diverter stent and the precise deployment of coils within the aneurysm. The duration of the procedure was approximately two hours. Following the endovascular procedure, the patient was transferred to the neurointensive care unit for close monitoring. The hospital course involved neurological assessments, including regular examinations of cranial nerve functions, motor and sensory evaluations, and the assessment of cognitive status. The patient's vital signs, including blood pressure and heart rate, were closely monitored to detect any signs of complications during the immediate post-procedural period.

During her hospital stay, the patient experienced a gradual resolution of her headaches. This positive response was accompanied by stability in neurological examinations. The absence of new neurological deficits and the alleviation of symptoms were promising signs of the success of the endovascular coiling procedure. The patient was discharged on clopidogrel and continued to do well. A thorough follow-up plan, including outpatient visits at specified intervals, was established to monitor the long-term outcomes and ensure the sustained well-being of the patient. The patient remained on clopidogrel 75 mg and aspirin 325 mg per day upon discharge, which formed part of a comprehensive and tailored post-procedural care plan.

## Discussion

Basilar artery aneurysms pose significant challenges due to the high risk of rupture and their impact on vital neurological structures within the brainstem and cerebellum. The anatomy of the basilar artery spans from the confluence of the vertebral arteries to the posterior cerebral arteries [1]. The pathophysiology of basilar artery aneurysms involves a multifaceted interplay of genetic predisposition, hemodynamic stress, and structural vulnerabilities in the arterial wall. Genetic factors, including familial clustering and hereditary connective tissue disorders, contribute to the basilar artery's susceptibility. Concurrently, hemodynamic stress and structural weaknesses in the arterial wall amplify the risk of aneurysm formation [1,4].

Historically, microsurgical techniques, including clipping and wrapping, have played a central role in managing basilar artery aneurysms [2]. Clipping involves the precise placement of a metallic clip around the aneurysm neck while wrapping employs biocompatible materials for mechanical support [2]. Intraoperative neurophysiological monitoring and neuroanesthesia play pivotal roles in ensuring surgical precision and patient safety [5].

The evolution of endovascular interventions, encompassing coil embolization, stent-assisted coiling, flow diversion, and the use of liquid embolic agents, heralds a transformative phase in basilar artery aneurysm management [3]. Endovascular techniques offer minimally invasive alternatives with reduced morbidity. Coil embolization involves the deployment of detachable coils within the aneurysm, while stent-assisted coiling and flow diversion provide supplemental support and redirect blood flow. The use of liquid embolic agents affords flexibility in managing challenging basilar artery aneurysm presentations. Neuroimaging guidance, including 3D rotational angiography and cone-beam CT, assumes paramount importance, ensuring precision during endovascular interventions [3,5].

While analyzing treatment outcomes of giant basilar artery aneurysms, it is crucial to consider findings from other relevant studies. A study by Wiebers et al. explored the controversial management of unruptured intracranial aneurysms [6]. The International Study of Unruptured Intracranial Aneurysms involved 4060 patients and revealed five-year cumulative rupture rates based on aneurysm size and location. Aneurysms in specific locations showed varying rupture rates, and the risks associated with surgical or endovascular repair often equaled or exceeded these rates. This highlights the complexity involved in managing patients with unruptured intracranial aneurysms, emphasizing the need to weigh natural history risks against those associated with the intervention.

Furthermore, Kiyofuji et al. conducted a meta-analysis on non-saccular aneurysms in the posterior circulation treated with flow diverters [7]. The analysis of 129 patients with 131 treated aneurysms demonstrated the feasibility and efficacy of flow diversion. However, it also highlighted a notable risk of periprocedural stroke and high overall mortality. Both microsurgical and endovascular interventions carry inherent risks. Neurological complications, vasospasm, intraoperative rupture, rebleeding, hydrocephalus, and postoperative infections represent critical facets requiring astute consideration. Postoperative care is pivotal and should encompass vigilant monitoring, rehabilitation, and ongoing support to address the impact on quality of life and functional status [3,5].

## Conclusions

This report of a giant basilar artery aneurysm sheds light on the challenges in managing such cases due to their potential life-threatening complications. The successful treatment and positive outcome in this instance demonstrate the significance of a multidisciplinary approach involving neurosurgery, interventional radiology, and critical care. Further research and continuous medical vigilance are warranted to enhance our understanding of the pathophysiology and optimal management strategies for giant basilar artery aneurysms, ultimately improving patient outcomes and quality of life. Additional studies involving long-term follow-up are necessary to gain deeper insights into optimal therapeutic strategies for giant basilar artery aneurysms.
